# Molecular Toxicology and Cancer Prevention

**DOI:** 10.3390/molecules28237730

**Published:** 2023-11-23

**Authors:** Guohui Sun, Chongwen Wang

**Affiliations:** 1 Beijing Key Laboratory of Environmental and Viral Oncology, Faculty of Environment and Life, Beijing University of Technology, Beijing 100124, China; 2 Laboratory Medicine, Guangdong Provincial People’s Hospital, Guangdong Academy of Medical Sciences, Guangzhou 510000, China

Molecular toxicology is a field that investigates the interactions between chemical or biological molecules and organisms at the molecular level. Chemical molecules, such as environmental organic contaminants and therapeutic drugs, are closely associated with the development of diseases such as cancer, and therapeutic effects [1,2,3,4,5,6,7,8]. Biomolecules, such as lncRNA, microRNA, and pathogenic proteins have important regulating effects on disease development [9,10,11,12]. In this Special Issue, we focus on the toxic effects and mechanisms of action of chemical and biological molecules. We collected 11 high-quality papers that will be of interest for researchers in molecular toxicology and applied pharmacology. Of the eleven papers, four papers are related to the environmental contaminants, four papers are related to biomolecules, two papers are related to natural organic small molecule products, and one paper is related to the application of biosensors in the detection of pathogenic microorganisms. We sincerely hope this Special Issue will prompt advances in molecular toxicology, as well its application in cancer prevention.

**Environmental contaminants.** The opening paper reported the effects of smoking on inflammatory-related cytokine levels in human serum [contribution 1]. In this study, Wang et al. [contribution 1] delved into the underlying mechanisms of cytokine production associated with tobacco smoking. A cytokine is a critical factor involved in the development of chronic systemic inflammation, which is the initial hallmark of cardiovascular and respiratory diseases as well as certain cancers. To explore this, blood samples from 78 male volunteers were categorized into three groups: non-smokers (30 individuals), current smokers (30 individuals), and ex-smokers (18 individuals). They used an advanced technique known as the liquid suspension chip method to analyze and compare the levels of 17 different cytokines and chemokines in the participants’ serum. The results unveiled distinct patterns in cytokine expression in response to smoking and smoking cessation. This study sheds light on the production of cytokines and chemokines under various smoking conditions, providing valuable insights into the early stages of smoking-related chronic diseases and cancers.

In the next paper, Sun et al. [contribution 2] focused on the impact of nicotine and a tobacco-specific carcinogen called 4-(methylnitrosamino)-1-(3-pyridyl)-1-butanone (NNK) on lung cells, particularly in the context of the cytochrome enzyme P450 2A13 (CYP2A13), which plays a role in metabolizing these substances. Past research has hinted at nicotine’s potential to reduce the harmful effects of NNK, but no comprehensive in vitro investigations have been conducted in lung cells, which are a key target organ for tobacco-related damage. To address this gap, they engineered a special cell line, BEAS-2B cells with a stable expression of CYP2A13 (referred to as B-2A13 cells), to explore how nicotine influences the cytotoxic and genotoxic effects of NNK. The results revealed that B-2A13 cells were more susceptible to NNK-induced cytotoxicity compared to regular BEAS-2B cells and control cells lacking CYP2A13 (B-vector cells). Notably, NNK induced significant DNA damage, caused cell cycle disruptions, and led to chromosomal damage in B-2A13 cells, whereas it had no significant impact on BEAS-2B cells or the control cells. This study demonstrates that CYP2A13 enhances the cytotoxic and genotoxic effects of NNK in BEAS-2B cells, and the presence of nicotine has the potential to mitigate the toxicity of NNK, providing valuable insights into the interplay between these substances in lung cells.

The presence of *Aspergillus flavus* and aflatoxins in grain crops poses a significant threat to food security and results in substantial economic losses. Aflatoxin B1, in particular, has been classified as a Group 1 carcinogen to humans by IARC. Traditional detection methods are often time-consuming and may not be as sensitive as required. However, Surface-Enhanced Raman Scattering (SERS) has emerged as a promising approach for the rapid and nondestructive detection of *Aspergillus flavus* and aflatoxins in grain crops due to its speed and sensitivity. Wang et al. [contribution 3] presented a rapid method for detecting *Aspergillus flavus* and quantitatively determining aflatoxin B1 in grain crops. This approach utilizes a portable Raman spectrometer in conjunction with colloidal gold nanoparticles (AuNPs). As the concentration of *Aspergillus flavus* spore suspension increased within the range of 10^2^–10^8^ colony-forming units per milliliter (CFU/mL), a more effective combination of *Aspergillus flavus* spores was observed and AuNPs resulted in an improved enhancement of the AuNPs solution’s effect on *Aspergillus flavus*. The authors successfully determined various concentrations of aflatoxin B1 in methanol solution by utilizing SERS, and the obtained spectra closely resembled those of solid powder samples. This rapid detection method has the potential to significantly reduce the detection time, from several hours or even tens of hours to just a few minutes. Such efficiency enables swift and effective measures to be taken to prevent substantial economic losses in the agricultural sector.

Polybrominated diphenyl ethers (PBDEs) are a class of both classic and emerging pollutants with the potential to harm the human immune system. Among PBDE congeners, 2,2′,4,4′-tetrabrominated biphenyl ether (BDE-47) stands out as the most biotoxic. In this study, Gao et al. [contribution 4] assessed the toxicity of BDE-47 on RAW264.7 cells, a type of mouse macrophage. The results reveal that exposure to BDE-47 resulted in a substantial reduction in cell viability and a notable increase in apoptosis. Furthermore, BDE-47 hindered the phagocytic activity of RAW264.7 cells, altered immune-related factors, and compromised immune function. Furthermore, a substantial increase in cellular reactive oxygen species (ROS) levels was noted. Transcriptome sequencing revealed the regulation of genes associated with oxidative stress. These findings strongly indicate that oxidative damage triggered by BDE-47 plays a pivotal role in mitochondrial apoptosis in RAW264.7 macrophages, ultimately resulting in the suppression of immune function. This study underscores the adverse effects of BDE-47 on immune cells and highlights the pivotal role of oxidative damage in producing these effects, with potential implications for understanding the immunotoxicity of PBDEs.

**Natural products.** Euphorbia factors are lathyrane-type diterpenoids found in the medicinal herb *Euphorbia lathyris L.* from the Euphorbiaceae family. These compounds have been associated with intestinal irritation toxicity, but the precise mechanisms behind this toxicity have remained a mystery. In the study by Zou et al. [contribution 5], they aimed to uncover the mechanisms by evaluating the transcriptome and microRNA (miRNA) profiles in human Caco-2 colon cancer cells after exposure to Euphorbia factors L1 (EFL1) and EFL2. The research protocol entailed subjecting Caco-2 cells to a 200 μM EFL treatment for a duration of 72 h. In the EFL1 group compared to the control group, there were alterations in the expression of 16 miRNAs and 154 mRNAs. Similarly, in the EFL2 group compared to the control group, 47 miRNAs and 1101 mRNAs exhibited differential expression. An analysis of these sequenced mRNAs highlighted their association with various critical processes, including transcription, post-translational modification, chaperones, protein turnover, secretion, vesicular transport, signal transduction mechanisms, intracellular trafficking, and cytoskeletal functions. The functions and pathways associated with differentially expressed mRNAs were enriched in areas such as transmembrane transport, T cell extravasation, the IL-17 signaling pathway, apoptosis, and the cell cycle. Additionally, the study identified that EFLs induced alterations in gene structures, encompassing processes like alternative splicing, insertion and deletion events, and single nucleotide polymorphisms. These findings offer valuable insights into the mechanisms that underlie the intestinal toxicity induced by EFLs in intestinal cells.

Conditionally replicating adenoviruses (CRAds) are a type of oncolytic adenovirus designed for tumor-targeted therapy. They can selectively enter cancer cells through coxsackievirus-Ad receptors (CARs), allowing them to replicate and destroy cancer cells while sparing normal cells. However, not all tumor cells express high levels of CARs, which can limit the effectiveness of CRAd therapy. Lu et al. [contribution 6] explored the use of 6-cyclohexyl methyl-β-D-maltoside (6-β-D) as a maltoside transfection agent. They found several advantages to using 6-β-D, including high transfection efficiency, low toxicity, and ease of use. By pretreating cancer cells with a low concentration of 6-β-D (≤5 μg/mL), they achieved an 18-fold improvement in the transduction efficiency of a “model” adenovirus (eGFP-Ad) compared to using eGFP-Ad alone. The addition of 6-β-D not only enhanced the transduction efficiency of CRAds but also improved their anti-tumor effects. Importantly, this combination did not harm normal cells. With 6-β-D treatment, CRAd at a lower multiplicity-of-infection ratio of 10 (MOI 10) achieved oncolytic outcomes similar to using a higher MOI of 50. This suggests that by combining CRAd with 6-β-D, the amount of CRAd required in clinical practice could be significantly reduced without compromising its therapeutic efficacy or exposing patients to potential side effects associated with high CRAd titers. This study provides a promising approach to improve adenovirus-mediated cancer gene therapy in clinical practice.

**Biomolecules.** The role of the neuroblast differentiation-associated protein AHNAK in biological processes has remained somewhat enigmatic. AHNAK is known to exhibit both suppressive and progressive functions in different types of cancers. In this study, Li et al. [contribution 7] sought to investigate the specific role of AHNAK in hepatocellular carcinoma (HCC). To understand its role, they conducted cell viability assays to assess the impact of AHNAK knockdown on cell proliferation in a stable HepG2 cell line. Additionally, co-immunoprecipitation (Co-IP) and LC-MS/MS were employed to analyze proteins in both HCC and matched paracancerous (MPC) tissues. The study revealed that silencing AHNAK led to a reduction in the viability of HepG2 cells. The analysis of protein interactions in HCC and MPC tissues identified 204 enriched pathways and processes. Furthermore, the study showed that AHNAK can co-localize and interact with the insulin-like growth factor 1 receptor (IGF-1R). This study sheds light on the involvement of AHNAK in HCC, emphasizing its potential contribution to HCC growth by interacting with IGF-1R.

Gene silencing is a crucial strategy in biology for understanding gene functions, investigating disease mechanisms, and developing potential therapeutics. The 8–17 DNAzyme is a promising tool for gene silencing due to its strong RNA-cleaving activity. However, its practical use has been limited by its dependence on divalent cations and a lack of comprehensive understanding of its cellular mechanisms. Zhou et al. [contribution 8] have investigated the activity of the 8–17 DNAzyme both in vitro and within cells. They discovered that this DNAzyme can effectively cleave RNA substrates under conditions that simulate physiological environments. Furthermore, its gene-silencing activity is enhanced by its compatibility with RNase H, allowing it to provide both cleavage and antisense activities within cells. They also found that chemical modifications can improve the stability, substrate binding affinity, and gene-silencing activity of the 8–17 DNAzyme. These findings suggest that this DNAzyme exhibits significant activity in cells, making it a valuable tool for exploring various biomedical applications.

Liu et al. (contribution 9) have introduced a novel prognostic signature based on aging-related long non-coding RNAs (lncRNAs) that is associated with immune cell infiltration and responses to breast cancer (BC) immunotherapy. They obtained BC samples from the breast-invasive carcinoma cohort within The Cancer Genome Atlas (TCGA) database and identified differentially expressed aging-related lncRNAs (DEarlncRNAs) using Pearson correlation analysis. Their analysis of the TCGA cohort led to the identification of a six aging-related lncRNA signature, comprising MCF2L-AS1, USP30-AS1, OTUD6B-AS1, MAPT-AS1, PRR34-AS1, and DLGAP1-AS1. The time-dependent ROC curve demonstrated the model’s strong prognostic predictability for BC patients. Patients in the low-risk group exhibited better overall survival and lower total tumor mutational burden. Additionally, the high-risk group had a smaller proportion of tumor-killing immune cells. It was also noted that the low-risk group derived more significant benefits from immunotherapy and specific chemotherapeutics compared to the high-risk group. This aging-related lncRNA signature offers new insights and strategies for early BC diagnosis and potential therapeutic targets, particularly in the context of tumor immunotherapy. We also published a review paper by Sumneang et al. [contribution 10], related to the toll-like receptor 4 (TLR4) inflammatory perspective on doxorubicin (Dox)-induced cardiotoxicity. In this review, the authors provide an overview of the existing evidence that supports the involvement of the TLR4 signaling pathway in various models of Dox-induced cardiotoxicity. They also discuss the impact of the TLR4 signaling pathway on Dox-induced cardiotoxicity. Gaining a better understanding of the role of the TLR4 signaling pathway in Dox-induced cardiac inflammation can aid in the development of therapeutic strategies to mitigate Dox-induced cardiotoxicity. This knowledge could be instrumental in the development of potential therapeutic approaches to address Dox-induced cardiotoxicity and improve the overall safety and efficacy of cancer treatment with Dox.

**Biosensors.** In this Special Issue, we published an interesting paper related to the graphene oxide (GO)-sensitized surface plasmon resonance (SPR) biosensor of porcine reproductive and respiratory syndrome virus. In this study, Liu et al. [contribution 11] investigated the impact of GO modification on the sensitivity of an SPR biosensor and utilized this GO-modified sensor for the detection of the porcine reproductive and respiratory syndrome virus (PRRSV) in cell cultures. The results revealed that GO modification significantly enhanced the sensitivity of the Fourier-transform SPR sensor. The GO-modified sensor, therefore, presents a promising alternative for virus detection. This study demonstrates the potential of GO-modified SPR biosensors for highly sensitive and label-free virus detection, paving the way for applications in virus diagnostics and research in biological samples.

To date, the toxicity of many chemical molecules and hypothetic molecules to special targets can be predicted by structure-based approaches like quantitative structure/activity/toxicity relationship (QSAR/QSTR), machine learning and artificial intelligence [1,2,4,5,8]. In addition, their toxic mechanisms can be further understood following advancements in bioinformatics, genomics, and proteomics. This Special Issue reported the toxic effects and mechanisms of four environmental contaminants and two natural products, as well as the role of three biomolecules in cancer development, gene silencing, and cancer prognosis. Additionally, the analytic determination of GO-based biosensor in pathogenic microorganisms was also reported. It should be noted that these advances represent a mere glimpse into the realm of molecular toxicology research. We sincerely hope that the published articles can inspire researchers in the field and stimulate further valuable investigations. Science is an evolving process, with progress made through affirmation and negation. Therefore, the conclusions and viewpoints of all articles purely reflect the outcomes under specific conditions. We look forward to seeing more valuable research in the field of molecular toxicology with the aid of this Special Issue, contributing to the advancement of science and the enhancement of human health.
